# Clinical Determinants of Disease Progression in Amyotrophic Lateral Sclerosis—A Retrospective Cohort Study

**DOI:** 10.3390/jcm10081623

**Published:** 2021-04-12

**Authors:** Maria Viktoria Requardt, Dennis Görlich, Torsten Grehl, Matthias Boentert

**Affiliations:** 1 Department of Neurology with Institute of Translational Neurology, Münster University Hospital (UKM), 48149 Münster, Germany; m.requardt@uni-muenster.de; 2 Institute for Biostatistics and Clinical Research, Münster University Hospital, 48149 Muenster, Germany; dennis.goerlich@ukmuenster.de; 3 Department of Neurology, Alfried Krupp Hospital, 45131 Essen, Germany; 4 Department of Medicine, UKM Marienhospital Steinfurt, 48565 Steinfurt, Germany

**Keywords:** amyotrophic lateral sclerosis, phenotypes, prognostic factors, disease progression

## Abstract

Background: Amyotrophic lateral sclerosis (ALS) is a neurodegenerative disease that is ultimately fatal but characterized by substantial phenotypic heterogeneity, which is known to impact long-term course and survival. This study investigated clinical determinants of disease progression and outcome in a large cohort of patients with ALS. Methods: Retrospective analysis included comprehensive data from 625 patients who attended a tertiary ALS centre at least twice. Patients were stratified according to five distinct clinical phenotypes: classical ALS; bulbar ALS; ALS with frontotemporal dementia (ALS-FTD); upper motor neuron predominant (UMNP); and lower motor neuron predominant (LMNP). Results: This study confirmed higher age at symptom onset, shorter latency to diagnosis and more rapid decline in the revised ALS Functional Rating Scale sum score as predictors of poor prognosis. Hazard ratios for shorter survival were higher in patients with ALS-FTD versus classical ALS, and in patients with versus without chronic obstructive pulmonary disease (COPD). Mean survival was longest in the UMNP phenotype group. Conclusions: This study confirmed established predictors of shorter survival in ALS and showed that concomitant COPD in particular relates to poor outcome.

## 1. Introduction

Amyotrophic lateral sclerosis (ALS) is a neurodegenerative disease characterized by involvement of both upper and lower motor neurons [1]. Neurodegeneration may affect the pyramidal tract or the anterior horn cells on different bulbar and spinal levels, resulting in a variety of clinical phenotypes. Both spasticity and hyperreflexia may be present in conjunction with muscle fasciculations, atrophy and weakness, affecting nearly all skeletal muscles [1,2]. With a lack of curative treatment options, progression is inevitable, resulting in tetraplegia, bulbar or pseudobulbar palsy, and chronic hypercapnic respiratory failure [3]. Median survival has been reported to be 2.5–5 years after symptom onset with substantial variability between different studies [3,4,5,6,7]. Premature death is mostly due to respiratory muscle weakness, aspiration, and cachexia [8,9]. Disease prevalence has been reported to be 5–8:100,000 [10,11], and symptom onset peaks at around 64 years of age [4,10].

Although ALS eventually leads to premature death in the vast majority of patients [3,4], various disease characteristics may show substantial inter-individual variability, including the site of initial symptom manifestation, the pattern of motor system involvement, and the speed of symptom progression [12]. Both patients and treating clinicians want to be able to estimate prognosis, attenuate disease progression, and anticipate critical events that require medical intervention and possibly impact prognosis. Several publications have consistently found older age, bulbar onset of disease, and faster symptom progress to indicate shorter survival [13,14,15]. In addition, distinct ALS phenotypes have been associated with better overall prognosis or slower disease progression [16]. However, rather few studies systematically focused on the clinical phenotype as a prognostic factor for disease progression and outcome [13,17,18,19,20]. For phenotypic classification, six to eight distinct entities have been proposed, including a “classical” (or Charcot’s) phenotype along with the following phenotypes: bulbar, flail arms, flail legs, respiratory, upper motor neuron predominant (UMNP), pure upper motor neuron (pUMN), pure lower motor neuron (pLMN), and lower motor neuron predominant (LMNP) [20]. This concept is theoretically limited by the fact that region of symptom onset and anatomical site of neurodegeneration are combined, possibly leading to some overlap. Only one study used a simplified set of phenotypes encompassing ALS (with bulbar, cervical or lumbar onset), flail limb syndrome, and pUMN disease [17]. None of these studies recognized ALS with frontotemporal dementia (ALS-FTD) as a distinct entity.

Therefore, the present study aimed to operationalize the above phenotypic classification. In addition, the prognostic impact of different phenotypes was investigated by taking into account established measures including age at symptom onset, weight loss, comorbidities, and functional decline on the revised ALS Functional Rating Scale (ALSFRS-R) [21].

## 2. Materials and Methods

### 2.1. Study Population

This retrospective single-centre study was approved by the local ethics committees regarding acquisition of clinical data and contact with patients or their descendants (Ethics committees of Münster University/Ärztekammer Westfalen-Lippe, Bochum University, and Ärztekammer Nordrhein). Patients were recruited from the University of Bochum ALS outpatient specialty clinic that moved to the Alfried Krupp Hospital in Essen, Germany, in 2016. All patients were seen by a single physician and investigator (T.G.). Inclusion criteria were informed consent, first appointment between January 2012 and July 2020, diagnosis of definite, probable (including probable laboratory-supported) or possible ALS according to the revised El-Escorial Criteria [22], and a minimum of two clinical visits. Follow-up visits were routinely scheduled every 3 to 6 months.

Out of 1173 individual patients who had presented during the above period, 62 were excluded because of missing consent. Follow-up data were lacking in 349 patients (single clinical consultation), and 137 subjects did not fulfil diagnostic criteria for ALS. Therefore, 625 patients were included in the study [23].

### 2.2. Data Collection and Patient Follow-Up

Data were continuously collected using FileMaker© Pro 17 Advanced software (Claris, Munich, Germany). The following data were obtained from medical records: age at symptom onset; age at diagnosis; clinical phenotype at first presentation; and diagnostic latency (time from symptom onset to diagnosis). In addition, the ALSFRS-R sum score and body mass index (BMI) at first and last clinical consultation were extracted. Lastly, family history, comorbidities, medication, and medical interventions specifically related to ALS, such as non-invasive ventilation (NIV), percutaneous endoscopic gastrostomy (PEG) or tracheostomy, were specifically recorded. The final BMI value was not available for three subjects, who were already bedridden at the last consultation. To increase completeness of data regarding medical interventions and current health status or date of death in particular, follow-up questionnaires were sent to patients or their authorized descendants.

The ALSFRS-R is an extensively validated 12-item scale assessing motor impairment with regard to bulbar function, hand and arm function, walking/gross motor abilities, and respiration [21]. The monthly decline of the ALSFRS-R sum score (‘slope’) has widely been used for monitoring disease progression and also predicts survival [15,24]. In order to account for any change of the progression rate over time, we calculated the early and late ALSFRS-R slope from data collected at the first and last clinical visit along with monthly decline of BMI during the same interval:

Early slope = (48 − ALSFRS-R at first consultation)/time from symptom onset to first consultation

Late slope = (ALSFRS-R at first consultation − ALSFRS-R at last consultation)/follow-up time in months)

BMI loss = (BMI at first consultation − BMI at last consultation)/follow-up time in months

A subset of patients had been diagnosed elsewhere and initially presented to our center with substantial delay. Therefore, we considered the possibility that disease progression from an unknown status would hamper reliable assessment of functional decline and BMI loss prior to the first visit. For better comparability with the rest of the cohort, we chose to analyze ALSFRS-R and BMI data only for patients who had presented to the outpatient clinic within the first six months after diagnosis (*n* = 456). In 169 patients, timespan between initial diagnosis and first consultation was longer (with a maximum of 132 months). However, all 625 patients were included in statistical analyses for all other measures captured at the initial visit.

Patients were stratified for age at symptom onset, and the early and late slope of the ALSFRS-R score (fast progressors: >1.11 points/month; intermediate progressors: 0.47–1.11 points/month; and slow progressors: <0.47 points/month [24]). In addition, patients were categorized according to the BMI (underweight: <18.5 kg/m^2^; normal weight: 18.5–24.9 kg/m^2^; overweight: 25.0–29.9 kg/m^2^; obese ≥ 30 kg/m^2^) [25].

**Figure 1:**
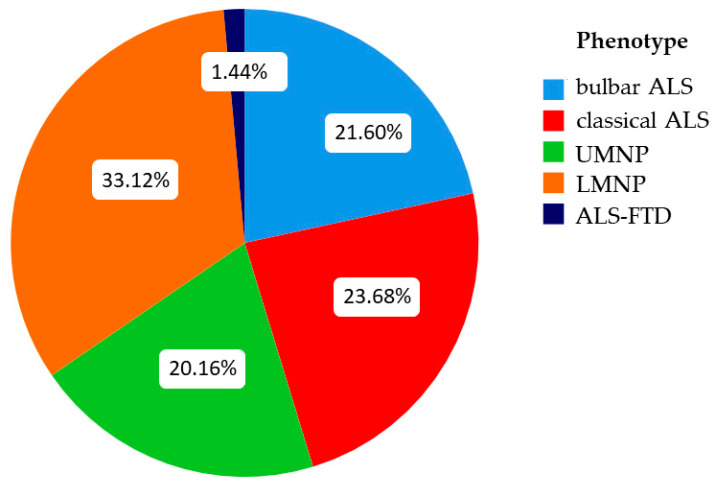
Distribution of clinical phenotypes. UMNP = upper motor neuron predominant; LMNP = lower motor neuron predominant; ALS-FTD = ALS with frontotemporal dementia.

In the original database, seven clinical ALS phenotypes were recognized and documented at the first clinical visit: bulbar, classical, UMNP, LMNP, flail arms, flail legs, and ALS-FTD. Phenotypic categorization of patients was based on the clinical and electromyographical findings on initial consultation. The bulbar phenotype was defined by bulbar or pseudobulbar onset and ongoing predominance of dysphagia or dysarthrophonia. The classic phenotype was ascribed to patients with initial onset of predominantly lower motor neuron (LMN) symptoms in two or more body regions in conjunction with clear but not predominant upper motor neuron (UMN) involvement as reflected by pyramidal signs or hyperreflexia. Predominant but not exclusive involvement of either UMN or LMN was classified as UMN predominant (UMNP) or LMN predominant (LMNP) disease, respectively. To take into account that the flail arms (27 patients) and flail legs (13 patients) phenotypes show preponderant degeneration of anterior horn cells, we chose to combine these entities with the LMNP group. Of note, this group also included patients who exhibited the pure LMN phenotype without any signs of UMN involvement (i.e., progressive muscular atrophy). Accordingly, patients exclusively showing signs of UMN degeneration (i.e., progressive lateral sclerosis) were assigned to the UMNP subgroup. Patients were assigned to the ALS-FTD phenotype when they had presented with predominant ALS while also fulfilling diagnostic criteria for frontotemporal dementia (FTD) [26], and in whom the ALS component was persistently salient. Patients with an isolated FTD had not presented to the outpatient clinic. In summary, five phenotypic categories were used for further data analysis: classical, bulbar, UMNP, LMNP, and ALS-FTD (Figure 1).

### 2.3. Statistical Analysis

All tests were conducted using IBM^®^ SPSS^®^ Statistics for Windows, Version 27.0 (IBM, Armonk, NY, USA). The Chi-square test was used to compare categorical variables, followed by post hoc tests with Bonferroni adjustment. One-way ANOVA was used for between-group comparison of age at symptom onset (normally distributed) and the Kruskal–Wallis test was applied for all non-categorical variables. Spearman-Rho was used to examine non-parametric correlations between metric variables. The Mann–Whitney U-test was utilized to evaluate gender difference, the McNemar–Bowker Test was used for subgroups based on the ALSFRS-R slope, evaluating group shifts in the entire cohort and within different phenotypes over time.

Overall survival was analyzed from symptom onset until death of any reason. Survival analyses were performed using Kaplan–Meier curves and the log-rank test for categorical variables, and univariable Cox regression for continuous measures. Finally, a multivariable Cox regression model was fitted for all variables that showed significance in the previous post hoc tests or univariable Cox analysis. First, a forward selection model (exclusion at *p* ≥ 0.1, inclusion at *p* ≤ 0.05) was conducted followed by backward elimination in order to check for consistency.

## 3. Results

### 3.1. Cohort Characteristics

The cohort included 222 patients with definite, 341 with probable and 62 individuals with possible ALS according to the revised El Escorial criteria (Table 1). The male-to-female ratio was 1.4:1 (58.4% male and 41.6% female), and ALS or FTD in at least one first-degree relative was reported by 3.5% of patients (22/625).

Mean ALSFRS-R sum score was 38.7 ± 5.64 points initially and 26.3 ± 9.5 points at the last visit. A total of 31.1% of patients (*n* = 142) were classified as fast progressors for early slope, 40.8% (*n* = 186) as intermediate progressors, and 28.1% (*n* = 128) as slow progressors. According to the late slope, 53.1% of patients (*n* = 242) were in the fast-progressing group, while 28.5% (*n* = 130) were intermediate progressors, and 18.4% (*n* = 84) were slow progressors.

Intake of riluzole at any time during follow-up was reported for 551 patients (88.2%). Of these, 393 patients (62.9%) reported regular medication with riluzole until the last visit (for 16.6 months on average). One hundred and fifty-eight patients (33.3%) stopped riluzole during follow-up (median duration of intake: 9.3 months), and 76 patients did not specify the duration of riluzole administration.

According to the BMI recorded at the first visit, 53.5% patients (*n* = 244) had normal weight, 31.8% (*n* = 145) were overweight and 3.7% (*n* = 17) were underweight [25]. Obesity (BMI > 30 kg/m²) was observed in 11% (*n* = 50).

Arterial hypertension was the most frequent concomitant disease (*n* = 246; 39.4%). Chronic obstructive pulmonary disease (COPD) was reported by 3.8% of patients (*n* = 24). Additional comorbidity data are reported in Supplemental Table S1.

At last visit, use of NIV was reported by 21.9% (*n* = 137). Tube feeding (via PEG) was reported by 28.6% (*n* = 179) and tracheostomy had been performed in 3.0% of patients (*n* = 19).

### 3.2. Clinical Phenotypes

As described above, patients were stratified according to five predefined clinical phenotypes: bulbar, classical, UMNP (including the pUMN phenotype), LMNP (including the flail arms, flail legs and pLMN phenotypes) and ALS-FTD (Figure 1). Disease characteristics of the different clinical phenotype subgroups are shown in Table 2.

The bulbar phenotype showed significant female predominance, while the majority of patients in the LMNP subgroup were male (both *p* < 0.0001). Mean age at disease onset was higher in patients with the bulbar phenotype compared with the classical (*p* < 0.0001), UMNP (*p* = 0.013) or LMNP phenotype (*p* = 0.016). Mean age at diagnosis, however, was only significantly higher in bulbar versus classical phenotype group (*p* < 0.001). Diagnostic latency was longer in the LMNP group versus the bulbar (*p* = 0.001) and classical (*p* = 0.004) phenotype, and also longer for the UMNP subgroup compared with the bulbar phenotype (*p* = 0.027). Phenotype groups did not differ significantly with respect to BMI at first consultation, BMI loss during follow-up (Table 2), and ALSFRS-R sum score at first consultation.

**Figure 2:**
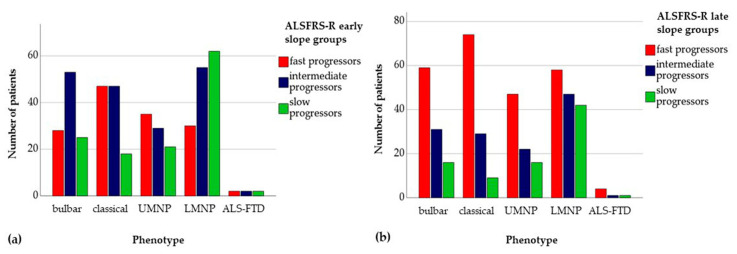
Distribution of ALS phenotypes according to disease progression rate as reflected by (**a**), ALSFRS-R early slope, and (**b**), ALSFRS-R late slope. ALS = amyotrophic lateral sclerosis; ALSFRS-R = revised ALS Functional Rating Scale; ALS-FTD = ALS with frontotemporal dementia; LMNP = lower motor neuron predominant; UMNP = upper motor neuron predominant.

The early slope of the ALSFRS-R sum score was significantly lower in the LMNP phenotype group compared with the bulbar (*p* = 0.014), classical (*p* < 0.0001) and UMNP (*p* = 0.001) phenotypes. The late ALSFRS-R slope was lower in the LMNP phenotype only compared with the classical phenotype (*p* < 0.0001). Distribution of slope categories within phenotype subgroups is depicted in Figure 2.

### 3.3. Early and Late Slope of ALSFRS-R

Both ALSFRS-R early and late slope were significantly higher in women than in men. Mean early slope was 1.05 in females and 0.93 in males (*p* = 0.007); mean late slopes were 1.51 and 1.07, respectively (*p* = 0.035).

Patients aged <61 years at symptom onset showed a significantly lower early slope compared with subjects who were older at the time of symptom onset (mean slope 0.93 vs. 1.03, *p* = 0.002). In contrast, the late slope did not differ significantly by age. Furthermore, it was statistically correlated with average BMI loss during the observational period (*r* = 0.35; *p* < 0.0001).

Patient grouping according to the ALSFRS-R slope changed during disease progression, resulting in significant differences between early and late slope group distribution in the McNemar–Bowker test (*p* < 0.0001). In the entire study population, 44.3% of patients maintained their initial slope group, while 41.5% changed into a faster progressing group, and 14.3% switched into a slower progressing group (Table 3). Bulbar, classical and LMNP phenotype groups showed a significant change in slope groups, while patients in the UMNP and ALS-FTD phenotype groups did not follow this trend (Table 3). There was a negative correlation between diagnostic latency and the ALSFRS-R early slope (*r* = 0.59).

### 3.4. Survival Analysis

**Figure 3:**
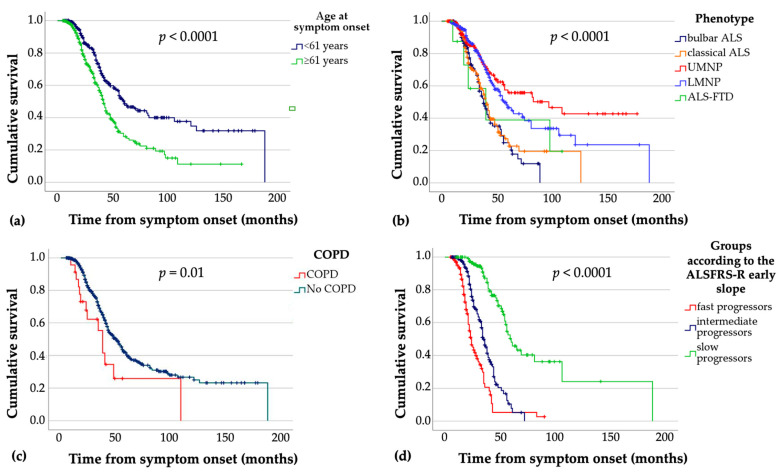
Kaplan–Meier survival curves according to (**a**) age at symptom onset; (**b**) clinical phenotype; (**c**) comorbid COPD; (**d**) ALSFRS-R early slope. ALSFRS-R = revised ALS Functional Rating Scale; ALS-FTD = ALS with frontotemporal dementia; COPD = chronic obstructive pulmonary disease; LMNP = lower motor neuron predominant; UMNP = upper motor neuron predominant. *p* values refer to the overall pooled log-rank (Mantel Cox) test.

In the entire study cohort, median survival was 48 months (first to third quartile, 32–121 months) from symptom onset. There were no significant sex differences. Survival differed significantly between groups when patients were stratified according to the initial phenotype, age at symptom onset (using the median of 61 years as cut-off), and the ALSFRS-R early slope (Table 4, Figure 3).

Shorter median survival was observed in the bulbar and classical phenotype groups compared with both the UMNP and LMNP phenotypes (both *p* < 0.001). In the UMNP subgroup, median survival was 59 months longer than in the bulbar ALS subgroup (Figure 3b).

While survival was not significantly different between males and females, older age at symptom onset (>61 years) was associated with shorter median survival compared with the younger group (by 1.5 years; *p* < 0.001) (Figure 3b). Furthermore, patients categorized as fast progressors (according to the early slope of the ALSFRS-R score) showed shorter median survival than intermediate and slow progressors (both *p* < 0.001, Figure 3d). Slow progressors also showed longer median survival compared with the intermediate progressor group (*p* < 0.001). In patients with concomitant COPD (24/625 patients, 3.8%), median survival was 13 months shorter than in patients without COPD (Figure 3c). Regarding BMI categories, no significant group differences were observed. Other comorbidities (hypertension, dyslipidemia, malignant disease, type 2 diabetes, depression, stroke, coronary artery disease) were not statistically associated with shorter survival compared with unaffected individuals.

Univariable Cox regression was performed for continuous variables. Age at symptom onset and the ALSFRS-R early slope, previously examined as categorical variables in the log-rank test, showed statistical significance in the Cox regression model (with hazard ratio (HR) values of 1.04 and 1.50, respectively; both *p* < 0.0001). Furthermore, shorter delay of diagnosis showed an association with shorter survival (HR 0.94, *p* < 0.0001). For BMI, statistical significance was narrowly missed (HR = 0.97, *p* = 0.056). There was no significant difference in median survival between patients who took riluzole continuously compared to those with only temporary or no riluzole intake.

### 3.5. Multivariable Cox Regression Model

All parameters that had shown statistical significance in the log-rank test or univariate Cox regression analysis (phenotype, age at symptom onset, early slope, diagnostic latency, and COPD) were included into a multivariable Cox regression model. First, a forward selection model (exclusion at *p* ≥ 0.1, inclusion at *p* ≤ 0.05) was conducted followed by backward elimination in order to check for consistency. Age at symptom onset and the ALSFRS-R early slope were included as steady variables, not groups, for precision purposes. All variables were kept in the model in order to improve prognostic validity. All the above parameters turned out to be significant independent predictors of shorter survival, with the highest HR values seen for concomitant COPD and ALSFRS-R early slope (Table 5). With classical ALS as the reference category, patients with either the UMNP and LMNP phenotype were likely to survive longer, whereas those with the ALS-FTD phenotype showed a markedly increased HR for shorter survival but statistical significance was missed (*p* = 0.062), most likely due to the small size of this subgroup (Table 5).

## 4. Discussion

This study comprehensively investigated determinants of survival in patients with ALS. The findings suggest that higher age at symptom onset, faster disease progression following disease manifestation, shorter delay to diagnosis and concomitant COPD predict shorter survival.

The prognostic impact of COPD has previously been reported [27]. In the present study, coincidence of ALS and COPD was associated with an almost threefold risk of dying within one year after symptom onset compared with ALS patients without COPD. Although it seems obvious that pre-existing lung disease puts patients at particular risk for early ventilatory failure when diaphragm weakness evolves [8], our observation highlights that patients with both ALS and COPD require increased monitoring for respiratory impairment.

Furthermore, this study confirms that the specific ALS phenotype has substantial impact on overall prognosis. Thus, knowledge on general characteristics and outcome features of distinct phenotypes helps clinicians to counsel patients and plan disease-related interventions. In detail, the present study allows for the following conclusions:

- Coincidence of ALS with FTD is rare but clearly relates to short survival.
- Among the more common phenotypes of ALS, bulbar manifestation is associated with the worst outcome.
- Patients with classical ALS show a relatively high early progression rate, and overall prognosis is similarly poor as in patients with bulbar ALS.
- In the LMNP group, progression rate is slowest in early disease stages but subsequently increases and relates to intermediate survival.
- Short or intermediate survival in the bulbar, classical and LMNP phenotype groups can be attributed to a higher proportion of patients in whom disease progression accelerates or remains stable.
- In patients exhibiting the UMNP phenotype, progression rate is relatively high following symptom onset but then slows down in a substantial proportion of patients, leading to the longest mean survival of all phenotype groups.

### 4.1. Comparison with Published Cohorts

The present results are consistent with published ALS cohorts with respect to several prognostic characteristics, including male preponderance [28,29], mean age at diagnosis [30] and mean diagnostic latency of approximately 13 months [19]. Prevalence of family history of ALS was lower than the 5.1% rate previously reported for familial ALS (fALS) in a large meta-analysis [31]. However, positive family history and fALS are not equivalent, making direct comparison difficult [2,31]. Similar to previous observations, we found that female patients showed higher early and late ALSFRS-R slopes than males, reflecting faster disease progression. Though, age at symptom onset, diagnostic latency, BMI at first consultation, BMI loss, and overall survival did not differ significantly between males and females. Contradictory information regarding the impact of sex on prognosis has been reported previously. While a few publications reported shorter survival in female patients [5,32], most publications agree on a similar prognosis for both sexes [15,33], which this study confirms. In the present study, patients in the LMNP group were significantly more likely to be male, whereas the majority of patients with bulbar ALS were female (male-female-ratio 0.69). These findings are in line with previous reports [10,19,20].

Regarding the distribution of distinct ALS phenotypes, the present work differs from existing population-based studies [19,20]. Whereas prevalence of the bulbar and classical phenotypes was lower compared with previous reports, the LMNP group included a higher proportion of patients than the above multicentric studies from Germany and Italy. The latter two separated the flail arm and flail leg phenotypes but, notably, the study by Wolf et al. did not recognize the pure LMN phenotype and showed a strikingly high percentage of patients with classical ALS (42.0% [19]) compared to the present study (23.7%) and data from Chiò et al. (30.3% [20]). These differences may, in part, be attributed to the fact that phenotypic categorization of patients was revised during follow-up in both previous studies whereas the initial classification was exclusively used in the present work.

### 4.2. Clinical Determinants of Prognosis and ALS Phenotypes

The present study confirms established prognostic factors in ALS [15,24,33,34,35,36]. A higher ALSFRS-R early slope [15,24,36], higher age at disease onset [15,33,34,35,37] and shorter diagnostic latency [15,33,34,35,37] have previously been identified as independent prognostic predictors of survival. In contrast, the present study did not statistically support previous reports on the prognostic impact of initial BMI [38]. However, for BMI at first consultation, statistical significance for prediction of survival was only narrowly missed. Most of the above factors were statistically different between distinct phenotypes, which had not been observed in smaller studies [19,39], but confirmed one previous large retrospective study [20].

In the bulbar phenotype group, patients were predominantly female and older at onset of symptoms. In addition, patients with bulbar ALS showed shorter diagnostic latency, suggesting that bulbar palsy is easier for clinicians to recognize than other manifestations of motor neuron disease. Most importantly, we were able to confirm previous reports that the bulbar phenotype has a particularly poor prognosis [15,33,34,35]. Regarding disease progression, the early slope of functional deterioration in bulbar ALS is similar to the UMNP and LMNP phenotypes, but almost half of patients with bulbar ALS show later acceleration of functional decline.

The classical ALS group showed one favorable characteristic (youngest mean age at onset), but two adverse features (steepest early slope of all groups and short diagnostic delay), resulting in a mean survival time that was only months longer than the bulbar group. This observation largely concurs with previous studies [19,20].

In the LMNP group, the majority of patients were male, as previously reported [19]. The early slope of the ALSFRS-R sum score was markedly lower than in any other phenotype which also confirms previous work [19]. As a result (and possibly due to higher similarity of clinical symptoms with other neurological conditions), delay of diagnosis was relatively long in patients with the LMNP phenotype. In patients with LMNP disease, late slope was higher than early slope, including a large number of patients (89.2%) for whom the progression rate was accelerated or at least retained. Notably, mean monthly decline on the ALSFRS-R worsened from the intermediate category (early slope) to the fast progression group (late slope). As a result, survival was only in the medium range and shorter than would have been expected based on ALSFRS-R early slope. This may reflect the impact of progressive motor impairment and phrenic nerve involvement at later stages.

Patients with the UMNP phenotype had a steep early slope (comparable to bulbar ALS), but the longest diagnostic delay and a younger age at onset. Although functional decline was relatively rapid early on, this group showed longer survival than all phenotype groups, which confirms previous observations [35]. Thus, more rapid progression of motor symptoms in early stages does not necessarily relate to shorter survival because it seems to be a unique feature of the UMNP phenotype that functional decline attenuates in later stages or does not correspond to earlier death, at least. We also believe that measurement of motor function may be limited in this particular phenotype by inherent features of the ALSFRS-R. More specifically, the type of central movement disturbance that evolves in patients with UMN-predominant ALS appears to result in rapid decline of the ALSFRS-R sum score early on but may not be adequately reflected by this scale’s items in later stages. In particular, UMN involvement itself does not result in respiratory muscle weakness that has a substantial impact on the ALSFRS-R sum score and life span [8,9,40]. This interpretation is strengthened by the fact that patients with UMN-predominant ALS, compared to patients with the classical, bulbar or LMNP phenotypes, more often switched to a slope group with slower progression rate. Similar to previous studies, the UMNP group showed a long delay to diagnosis, which was almost twice as long as that in patients with bulbar ALS. This appears to contradict the relatively high early progression rate, but may be attributed to the fact that patients with UMN-predominant disease often show “atypical“ clinical features or do not formally fulfil El-Escorial Criteria [2], especially in early disease stages.

Only nine patients with the ALS-FTD phenotype were included in the present study, and of those only six had their first visit within six months following diagnosis. Therefore, statistical conclusions are very limited regarding this subgroup. However, the findings reported here concur with previous work, regarding exceptionally short survival [19,41], but also long delay of diagnosis.

### 4.3. Strengths and Limitations

It is a strength of the present study that data acquisition was continuously performed by a single physician and ALS expert over a period of eight years. Thus, a systematic approach to data collection and documentation was used, yielding detailed and complete patient records. Furthermore, this study comprehensively took into account various disease characteristics that have been shown to determine long-term outcome in patients with ALS. Thirdly, categorization of ALS phenotypes aimed to provide a feasible set of phenotypes that primarily reflect the site of motor neuron degeneration (UMNP vs. LMNP vs. classical), but also recognize predominant bulbar involvement and ALS-FTD as clinically distinct entities. In two previous studies, ALS-FTD was not listed as a category on its own, which does not allow for specific conclusions with regard to the phenotype [19,20].

The present study also has several limitations. Firstly, the sample size (n = 625) is only intermediate when compared to previous studies that focussed on disease phenotypes [17,18,20]. However, it clearly exceeds studies applying a similar approach (Wolf et al. with 200 patients [19], Abdul Aziz et al. with 144 patients [42]). Secondly, this study did not specifically recognize the impact of home ventilatory support, tube feeding and tracheostomy on overall survival. Since clinical data were collected in an outpatient clinic (but with NIV initiation, gastrostomy and tracheostomy being inpatient procedures), relevant information was based on reports given by either the patients themselves or caregivers and descendants only. Even though we aimed to improve data quality by sending follow-up questionnaires, the number of medical interventions we were able to document was most likely lower than reality. NIV usage was 21.8% in the present work but has been reported to be 5–35% in European cohorts [30,43] and up to 41.1% in a previous study from Germany [28]. Gastrostomy was recorded for 28.6% of patients, coming close to numbers derived from different European multicentric studies [28,43]. Tracheostomy had been performed in 3% of the study population only, which could again be due to incomplete reporting, but highly variable numbers, ranging from 10 to almost 30%, have been reported previously [44,45]. The present study was not designed to specifically address the prognostic impact of NIV, PEG insertion or tracheostomy, and valid conclusions get even more difficult when different ALS phenotypes are taken into account. Finally, it may be seen as a limitation that 349 patients were excluded who attended the outpatient clinic only once and were then lost to follow-up. However, these individuals would have been censored immediately on survival analysis, and one can only speculate about the statistical impact of this cohort on the Kaplan–Meier curves if either phenotype distribution or survival had been systematically confounded.

### 4.4. Conclusions

This study confirmed established predictors of shorter survival in ALS including higher age at disease manifestation, faster disease progression following symptom onset, and shorter delay of diagnosis. In addition, this study highlights the prognostic impact of the specific ALS phenotype which has to be recognized in individual patient counselling. Patients with ALS and concomitant COPD bear a particular risk of premature death, and close respiratory monitoring is compulsory in this population.

## Figures and Tables

**Table 1:** General characteristics of the study population (*n* = 625).

Parameter (Unit)	Mean ± SD	Median (1st–3rd Quartile)
Age at symptom onset (years)	60.64 ± 10.17	61 (53–69)
Age at diagnosis (years)	61.81 ± 10.14	62 (54–70)
Diagnostic latency (months)	13.44 ± 14.99	8 (5–17)
Diagnosis to first consultation (months)	6.59 ± 11.69	3 (1–7)
BMI at first consultation (kg/m²)	24.87 ± 4.27	24.11 (22.13–26.98)
BMI loss (kg/m² per month)	0.20 ± 0.34	0.12 (0.00–0.32)
ALSFRS-R early slope (points/month)	0.98 ± 0.85	0.75 (0.42–1.3)
ALSFRS-R late slope (points/month)	1.40 ± 1.24	1.17 (0.6–1.75)
Total duration of follow-up (months)	14.03 ± 14.99	9 (4–17)

ALSFRS-R = revised ALS Functional Rating Scale; BMI = body mass index; SD = standard deviation.

**Table 2:** Clinical characteristics of different ALS phenotypes.

Parameter		Bulbar	Classical	UMNP	LMNP	ALS-FTD	*p*-Value
Number of patients		135	148	126	217	9	
Gender	M–F-Ratio	0.69	1.35	1.25	2.51	3.5	
	Female	59.3%	42.6%	44.4%	28.5%	22.2%	<0.0001 *
Age at symptom onset (years)	Mean	63.99	58.34	59.98	60.48	61.22	0.0001 ^#^
	Median	64	59	60.5	60	63	
Age at diagnosis (years)	Mean	64.81	59.21	61.56	61.81	63.00	0.0002 ^#^
	Median	65	59.5	62	62	64	
Diagnostic latency (months)	Mean	9.41	10.12	17.79	15.47	20.78	
	Median	7	7.5	10	10	9	0.0002 ^§^
Early slope (ALSFRS-R)	Mean	1.02	1.21	1.09	0.72	1.06	
	Median	0.79	0.98	0.81	0.57	0.8	<0.0001 ^§^
Late slope (ALSFRS-R)	Mean	1.32	1.67	1.43	1.25	1.28	
	Median	1.16	1.33	1.22	0.86	1.38	0.0003 ^§^
BMI at first visit (kg/m²)	Mean	24.97	24.66	26.04	24.31	23.93	
	Median	23.89	23.94	25.76	23.62	23.62	0.065 ^§^
BMI loss per month ((kg/m²)/month)	Mean	0.24	0.19	0.18	0.2	0.2	
	Median	0.21	0.13	0.09	0.11	0.2	0.122 ^§^

ALS-FTD = ALS with frontotemporal dementia; ALSFRS-R = revised ALS Functional Rating Scale; BMI = body mass index; LMNP = lower motor neuron predominant; UMNP = upper motor neuron predominant; * Chi-square test; ^#^ ANOVA; ^§^ Kruskal–Wallis test.

**Table 3:** McNemar–Bowker test for slope group consistency.

Group		Change to Faster Group	Stay in Same Group	Change to Slower Group	*p* Value
All patients		41.5%	44.3%	14.3%	<0.0001
Phenotype	Bulbar	46.2%	38.7%	15.1%	0.0002
	Classical	42.9%	43.8%	13.4%	0.0005
	UMNP	36.5%	43.5%	20%	n. s.
	LMNP	39.5%	49.7%	10.9%	<0.0001
	ALS-FTD	50%	33.3%	16.7%	n. s.

ALS-FTD = ALS with frontotemporal dementia; LMNP = lower motor neuron predominant; UMNP = upper motor neuron predominant.

**Table 4:** Median survival time from symptom onset.

Group/ Parameter	Subgroup	Median Survival in Months (1st–3rd Quartile)	Mean Survival (Months)	*p* Value (Log-Rank Test)
All patients		48 (32–121)	78.26	
Phenotype	Bulbar	38 (26–56)	44.27	<0.0001
	Classical	40 (24–61)	53.69	
	UMNP	97 (36–97)	103.35	
	LMNP	56 (38–121)	85.41	
	ALS-FTD	40 (20–98)	55.69	
Age at symptom onset	<61 years	61 (36–188)	94.94	<0.0001
	≥61 years	42 (26–72)	58.15	
Early slope (ALSFRS-R)	Fast	24 (18–35)	28.87	<0.0001
	Intermediate	35 (25–45)	37.49	
	Slow	60 (46–106)	89.92	
COPD	Yes (*n* = 24)	38 (18–109)	49.23	0.013
	No (*n* = 601)	51 (33–126)	79.85	

ALS-FTD = ALS with frontotemporal dementia; ALSFRS-R = revised ALS Functional Rating Scale; COPD = chronic obstructive pulmonary disease; LMNP = lower motor neuron predominant; UMNP = upper motor neuron predominant.

**Table 5:** Probability of survival (multivariable Cox regression model with forward inclusion).

Variable	Hazard Ratio	95% Confidence Interval	*p* Value
Phenotype			0.004
Classical	Reference category		
Bulbar	0.72	0.49–1.07	n. s.
UMNP	0.55	0.36–0.84	0.006
LMNP	0.62	0.42–0.92	0.017
ALS-FTD	2.65	0.95–7.35	n. s.
Diagnostic latency	0.94	0.93–0.96	<0.0001
ALSFRS-R early slope	1.33	1.19–1.5	<0.0001
Age at onset	1.04	1.03–1.06	<0.0001
COPD vs. no COPD	2.82	1.53–5.18	0.001

ALSFRS-R = revised ALS Functional Rating Scale; ALS-FTD = ALS with frontotemporal dementia; COPD = chronic obstructive pulmonary disease; UMNP = upper motor neuron predominant; LMNP = lower motor neuron predominant.

## Data Availability

The data presented in this study are available on request from the corresponding authors. The data are not publicly available due to legal restrictions.

## Supplementary Materials

The following are available online at https://www.mdpi.com/article/10.3390/jcm10081623/s1, Table S1: Most frequent comorbidities in the study cohort.
