# Supplementary material for: Clinical Determinants of Disease Progression in Amyotrophic Lateral Sclerosis—A Retrospective Cohort Study
Source: J Clin Med. 2021 Apr 12;10(8):1623. doi: 10.3390/jcm10081623 (PMC8069893; doi:10.3390/jcm10081623)
Supplement: Supplementary file 1 [file jcm-10-01623-s001.pdf]

Table S1. Most frequent comorbidities in the study cohort (n = 625)

Comorbidity	Number of patients	Percentage
Arterial hypertension	246	39.4%
Dyslipidemia	65	10.4%
Malignant disease	48	7.7%
Type 2 diabetes	32	5.1%
Depression	41	6.6%
COPD	24	3.8%
Coronary heart disease	27	4.3%
Stroke/TIA	21	3.4%
Other comorbidities	150	22.7%
No comorbidities	135	21.6%

COPD = chronic obstructive pulmonary disease; TIA = transitory ischemic attack.
